# Environmental factors that impact the eating behavior of home-living older adults

**DOI:** 10.1016/j.ijnsa.2021.100046

**Published:** 2021-10-12

**Authors:** Fifi Kvalsvik, Torvald Øgaard, Øystein Jensen

**Affiliations:** University of Stavanger, 4021 Stavanger, Norway

**Keywords:** Dyadic, Eating behavior, Ecological model, Health promotion, In-depth individual, Nutrition, Older adults

## Abstract

**Objective:**

To identify environmental factors that influence the eating behavior of home-living older adults.

**Design:**

Qualitative study with two interview methods, dyadic and in-depth individual interviews.

**Setting:**

The study was conducted in a western district of Norway.

**Participants:**

A total of 22 participants. The study sample consisted of 8 dyads for the dyadic interviews and 6 participants for the in-depth individual interviews. The dyads were composed of pairs who share a pre-existing relationship as well as pairs of strangers.

**Method:**

The qualitative study uses deductive and inductive content analysis.

**Results:**

Seven environmental factors that play a role in older adults' eating behavior were organized into three levels of influence: interpersonal influence (food habits of significant others, household composition, and social relationship), community influence (senior centers and food access), and public policy influence (health information and transportation/mobility aids).

**Conclusion:**

Various environmental factors determine the eating behavior of older adults living at home. An approach is needed to address these factors in order to bring positive change in the eating behavior of home-living older adults. The findings suggest that a social environment may be used to encourage healthy eating. Furthermore, increasing participation in a senior center, ensuring access to food, reducing ambiguity in diet and nutrition information, and increasing mobility support can help older adults maintain or develop healthy eating behavior.

**What is already known about the topic?**

• An important factor in improving healthy aging is adequate food and a nutritionally sound diet.
• Interventions designed to promote healthy eating yielded minor improvement or inconsistent results.
• The aging population will increase the strain on public finances and the welfare system.

**What this paper adds**

• This study demonstrates that the social environment and community have the potential to change older adults' eating behavior in a desirable direction.
• Consistent health information conveyed through various media or health professionals may encourage older adults to change their eating behavior.

## 1 Introduction

Older adults constitute a growing proportion of the global population. There are about 1 billion people aged 60 and over today, and this will be doubled by 2050 (World Health Organization, 2018). The annual population statistics for 2018 show that in Norway, the number of people in the age group 67–79 years has increased by 42%, and for those aged 90 and older the increase is 29% (The Norwegian Directorate of Health, 2018). Norway's population is aging rapidly, and it is anticipated that there will be more older adults than children in 2030 (Statistics Norway, 2020). This demographic shift means a greater burden of chronic disease, disabilities, and frailty for older people themselves, and also adds to the challenges in health care and other social resources (Dean et al., 2009; Poscia et al., 2018).

Given this situation, it is hardly surprising that a range of health promotion approaches are designed to support healthy eating in home-living older adults. Despite the importance of healthy eating and nutrition for aging adults, changes associated with aging and eating habits in early life (Elsner, 2002; Lumbers and Raats, 2006) may not be conducive to encouraging older adults to eat in a manner that meets the energy or protein requirement for older adults. Furthermore, most older adults consider their diet to be sufficiently healthy, and thus not requiring any changes (De Almeida et al., 2001).

Although many older adults consume a nutritionally adequate diet, there is mounting evidence of malnutrition risk in home-living older adults (Kozáková and Zeleníková, 2014; Söderhamn et al., 2012). The term "malnutrition" in this paper refers to insufficient dietary intake to meet energy or protein requirements in old age (Roy, 1994). Previous studies reported that the prevalence of malnutrition among European older adults living at home ranges from 13% to 30% (Kozáková and Zeleníková, 2014; Söderhamn et al., 2012). The Norwegian Directorate of Health (2020) stated that in 2018, 15.8% of older adults receiving health care at home are at nutritional risk, compared to 19.2% in 2019 and 18% in 2020. With just a 1% decrease in the rate of nutritional risk, coupled with the current demographic transition, malnutrition can become a pressing concern for society (ESPEN, 2019).

Although nutrition and healthier diets for older adults are currently on the agenda of policymakers in Norway, a significant gap still exists between recommendations by healthcare professionals and the services offered to many older adults (Norwegian Ministry of Health and Care Services, 2018). One of the main challenges related to nutrition is the lack of systematic nutritional measures. Systematic nutritional measures refer to the follow-up on individual nutritional needs in order to prevent malnutrition (Norwegian Ministry of Health and Care Services, 2018). In Norway, only 16% of older adults receive systematic nutritional screening; therefore, malnutrition is often both underdiagnosed and undertreated (Ihle-Hansen et al., 2011).

Malnutrition is a multifactorial problem, and effective intervention measures are needed (Ihle-Hansen et al., 2011). However, despite all efforts, interventions aimed at modifying older adults' eating behavior have yielded little improvement or inconsistent results (Poscia et al., 2018). This may be partly due to an inadequate understanding of the factors associated with eating behavior among older adults that need to be addressed in the interventions. Research has been conducted to identify these factors, but such research often focuses solely on the individual determinants of eating behavior, such as attitudes, beliefs, and preferences (Bukman et al., 2020; Kremers et al., 2004). Furthermore, interventions to change eating behavior also often focus on individual-level influences (Brug et al., 2008). The focus on changes at the individual level is an important step in changing behavior. However, eating behavior is not an individual choice that is disconnected from the environment in which an individual lives (Brug et al., 2008). Rather, the environment is a critical force that plays a role in enabling or limiting people's ability to make dietary choices that support their health and well-being (Belon et al., 2016). Research on the complex dynamic of the environmental factors that impact on eating behavior is therefore warranted (Belon et al., 2016).

The importance of environmental factors' contribution to healthier eating behavior has been described in previous studies, such as the study on the vital role family plays in eating behavior (Brijnath, 2011) and the study on health professionals' role in guiding the eating behavior of their patients (Team et al., 2017). With that said, findings from other contexts can only be used as a starting point. When it comes to eating behavior, cultural background plays an important role; people tend to stick to what they know, and tradition rather than choice dominates a person's food world (Rozin, 2007).

Our study seeks to fill this knowledge gap by identifying environmental determinants of home-living older adults' eating behavior. The term "environment" describes a range of contextual factors influencing home-living older adults' eating behavior (Raine, 2005). The aim is to identify the environmental factors that may have the greatest influence on the eating behavior of home-living older adults. This study contributes to the existing literature on the importance of healthy eating for older adults. The findings can also be used as a sound basis for public decision-making.

## 2 Conceptual framework

Research in this area has shown that multiple environmental and individual factors impact older adults' eating behavior and, ultimately, health outcomes (Story et al., 2002). In this study, we use the ecological model proposed by McLeroy et al. (1988) to highlight the environmental factors that may affect the eating behavior of home-living older adults.

This model assumes that appropriate changes in the environment will produce changes in individuals (McLeroy et al., 1988). Moreover, the ecological model has been used extensively to determine factors that influence eating and other food-related behavior (Holsten et al., 2012; Story et al., 2002). We therefore consider it a suitable framework for exploring the environmental determinants of the eating behavior of home-living older adults.

Based on this framework, five broad levels are considered to influence behavior: intrapersonal (individual) factors, interpersonal (social) factors, institutional factors, community factors, and public policy (societal) (McLeroy et al., 1988).

Intrapersonal factors refer to individual characteristics that influence behavior, such as knowledge, attitudes, skills, beliefs, and preferences, while interpersonal factors are related to formal and informal social networks, including family, friends, and peers. Institutional aspects include social institutions with organizational characteristics, and community factors concern relationships among organizations, institutions, and formal networks within defined boundaries. Finally, public policy refers to local, state, national laws, policies.

## 3 Methods

Eating behavior is often influenced by a complex interaction of different factors (Bukman et al., 2020). To unlock the complexity of the eating behavior among home-living older adults, we chose to explore this phenomenon using two interview methods. The section below presents the two data collection approaches.

### 3.1 Data collection approaches

*The first approach is the dyadic interview* (Morgan et al., 2013). The term "dyadic interview" refers to interviewing two participants together to collect valuable data for a research project (Morgan et al., 2013). We chose the dyadic method for two reasons. First, it takes into account the characteristics of old age that could threaten the validity of the data. A classic example of such characteristics is normal cognitive changes (Harada et al., 2013). As a result of changes in cognition, older adults may have a decline in processing speed, memory, and attention span (Harada et al., 2013). Dyadic interviews allow participants to have more time to process what has been said and formulate their responses (Morgan et al., 2013).

The second reason we chose the dyadic method was the nature of the research topic. Eating behavior is viewed as a complex phenomenon influenced by social context (Higgs and Ruddock, 2020). What we decide to eat and how we arrive at a decision is often a form of collaboration with others connected to us (Delormier et al., 2009; Higgs and Ruddock, 2020). Using dyadic interviews to explore a research topic related to collaboration can therefore contribute to the co-creation of new knowledge (Klevan et al., 2020). In other words, using dyadic interviews allows the content to be extended beyond what might have been possible in individual interviews (Morgan, 2016). Thus, the dyadic interview is likely a suitable method for collecting data from home-living older adults.

*The second approach is the in-depth individual interview*. The in-depth individual interview was selected because it represented the most widely used data collection method in qualitative studies (Wilson et al., 2016). The in-depth individual interview allows a researcher to explore a phenomenon from an individualistic perspective (Ryan et al., 2009). Moreover, it has often been argued that in-depth individual interviews tend to reveal more detailed information than other methods (Agar and MacDonald, 1995).

### 3.2 Participants

Definitions of the term "older adult" differ in the literature. One way of measuring old age is using a fixed chronological age without regarding how healthy a person is, how a person functions, or whether a person is actively working or retired (Scherbov and Sanderson, 2016). For the purpose of this study, we defined an older adult as a person aged 60 and over. Although we agree that chronological age is not the best predictor variable, it is the most common way to measure age (Skirbekk et al., 2019).

With that said, the older population comprises older adults with considerably different characteristics, so it is likely that people with certain characteristics, for example in relation to health and physical strength, are being excluded from research. People with a specific characteristic, however, are often the ones in most need of research to improve their condition (Sin, 2005). On this basis, we included a wheelchair user (58 years old) who is a member of a senior center in the study sample.

What follows is the nature of the research topic. Food choice is a complex construct that often involves other people connected to us (Higgs and Ruddock, 2020). To examine food choice as a construct and its variation, we therefore included younger participants in our parent-child dyad.

### 3.3 Sampling method

Our initial recruitment strategy entailed placing flyers in the mailboxes at senior housing complexes. The gatekeepers were informed of the study, and interested participants were instructed to contact the researchers by phone. We were, however, unable to recruit enough participants within the expected time frame using only this strategy. To overcome this issue, we adopted a more proactive recruitment strategy, in which we recruited participants from senior centers in the district. This approach involved a 30-minute presentation of our project to the members of activity centers. Subsequently, those interested in participating were asked to arrange a time and place for an interview. As time progressed, we employed street-intercept recruitment strategies in public places such as public libraries, coffee shops, and shopping centers to increase the number of participants. Those who agreed to participate were given the option of an individual interview or to be paired up with someone else.

To gain as broad understanding as possible into home-living older adults' eating behavior, we chose a sample of older adults who varied in age, gender, occupation, employment status, marital status, and living situation. In addition, for dyadic interviews, participants were paired together based on different types of relationships (see Table 1). We expected the variety of participants to enable us to capture variable perspectives of the phenomenon being studied (Suri, 2011).

**Table 1:** Description of participant characteristics from dyadic interviews.

Dyad pairs	Gender	Age	Occupation	Employment status	Marital status	Living situation
Married couple 1	Female Male	64 66	Teacher & counselor Engineer	Full-time Retired	Married	Living with a spouse
Married couple 2	Female Male	62 65	Manager at a kindergarten Substance abuse-related psychiatrist	Part-time Full-time	Married	Living with a spouse
Father-Son	Male Male	60 28	Counselor in an office Teacher	Full-time Full-time	Divorced In a relationship	Living alone Living with a partner
Mother-Daughter	Female Female	86 58	Shopkeeper Teacher	Retired Full-time	Widow Divorced	Living alone Living alone
Friends pair 1	Female Female	88 83	Tour guide Travel agency	Retired Retired	Widow Widow	Living alone Living alone
Friends pair 2	Male Male	80 76	Engineer Engineer	Retired Retired	Married Divorced	Living with a spouse Living alone
Strangers pair 1	Male Male	72 69	Civil engineer Engineer	Retired Retired	Married Single	Living with a spouse Living alone
Strangers pair 2	Female Male	63 60	Housewife Teacher	Unemployed Full-time	Married Married	Living with a spouse Living with a spouse

As a result, we recruited 22 participants, all of whom were Norwegian. Of these, 16 completed a dyadic interview, and 6 completed an in-depth individual interview. Of the 16 participants in the dyadic group, 8 dyads were established; 2 married couple dyads, 2 parent-child dyads, 2 friend dyads, and 2 stranger dyads.

The participants' characteristics are presented in Tables 1 and 2.

**Table 2:** Description of participant characteristics from in-depth individual interviews.

Participants	Gender	Age	Occupation	Employment status	Marital status	Living situation
Participant 3	Female	82	Housewife	Unemployed	Widow	Alone
Participant 8	Female	58	Housewife	Unemployed	Married	With spouse
Participant 13	Female	92	Housekeeper	Retired	Widow	Alone
Participant 16	Male	88	Businessman	Retired	Widow	Alone
Participant 17	Male	71	Skipper	Retired	Widow	Alone
Participant 20	Male	71	Petroleum engineer	Retired	Single	Alone

### 3.4 Data collection

The data collection took place between October 2019 and January 2020 in a district of Western Norway with approximately 482,000 inhabitants, including 100,100 older adults aged 60 and over (Statistics Norway, 2020). The study was reviewed and approved by the Norwegian centre for Research Data (2019/ 502,106), and written informed consent was obtained from all the participants individually before initiating the data collection. The interviews were held at a time and place of the participants' choice, including participants' homes, cafeterias at senior centers, libraries, and coffee shops. Each dyadic interview session lasted between 50 min to 1.5 h, while each session in an in-depth individual interview lasted about 40 min to 1 hour. Field notes were taken while conducting the interviews and all the interviews were audio-taped.

### 3.5 Data analysis

Upon completion of each interview, the audio files were listened to several times, and verbatim transcriptions were prepared for each interview. To ensure the accuracy of the transcriptions, the same datasets were also transcribed by a transcriber who was not involved in the data collection.

The data collected were analyzed using content analysis. Content analysis examines data with a view to understanding the meaning behind it (Krippendorff, 2018). As a research technique, it enables researchers to organize and elicit meanings from the data collected and draw a realistic conclusion (Bengtsson, 2016). Furthermore, content analysis offers the potential for making replicable and valid inferences of the data based on the overall context (Krippendorff, 2018).

The content analysis was inspired by method descriptions, primarily that of Elo and Kyngäs (2008) but also Hsieh and Shannon (2005). The data analysis was conducted in two distinct phases: deductive and inductive. This integrative methodological approach has so far not been extensively described in the nursing literature (Sandström et al., 2015). However, this approach is useful when a prior theory exists (here the ecological model) about a phenomenon (Hsieh and Shannon, 2005) and when knowledge about the phenomenon is still fragmented (Elo and Kyngäs, 2008).

#### 3.5.1 The deductive content analysis

We started this phase by selecting the unit of analysis. The unit of analysis in this study was a sentence that could be used to answer the research question ("what environmental factors may have the greatest influence on the eating behavior of home-living older adults?"). The next step in the analytical process was to make sense of the data collected. Here, each transcript was read several times thoroughly. Our aim is to become immersed in the data (Polit and Beck, 2004) and obtain a sense of whole (Burnard, 1991).

In the next phase, we developed a structured categorization matrix (Elo and Kyngäs, 2008) based on the ecological framework for health promotion (McLeroy et al., 1988). This categorization matrix was structured according to the different levels of influence considered to impact eating behavior. The categorization matrix was then used as a lens to analyze the data when we read through it again. The text corresponding to the categorization matrix was highlighted, coded, and transferred into the relevant categories in the matrix by the first author. The codes and their definitions were derived from previous qualitative food studies conducted with older adults, for example, food-related habits, food environment, and health information-seeking behavior (Bloom et al., 2017; Shim et al., 2019; Turner et al., 2018).

The texts that were considered not to fit into the matrix were saved in a separate document. Finally, when this was done for all the transcripts, the co-authors appraised the matrix, and all three authors appraised the document containing the "non-fitting" texts. This appraisal involved excluding the "non-fitting" texts that were deemed not relevant to the study (e.g., a participant's holiday plan) and transferring the remaining undecided texts to the matching category.

#### 3.5.2 Inductive content analysis

The aim in this phase is to gain a comprehensive understanding beyond the earlier categorization. To allow the participants' perspectives, experiences, and interpretations to emerge, we employed a qualitative approach inspired by grounded theory (Strauss and Corbin, 1990). With that said, the present study does not follow the pure inductive form as it begins with a pre-determined category derived from existing theory.

The transcripts were reread, and then texts were abstracted into codes. We used in vivo codes as much as possible to highlight the voices of our participants (Manning, 2017). The codes were developed separately by two authors to increase the comprehensivity and ensure sound interpretation (Schreier, 2012). Any disagreements on code descriptions were resolved through discussions among the authors during project meetings. Thereafter, the codes were structured into sub-categories based on their similarities and differences. Through the identification and interpretation of similarities and differences, further abstraction was achieved, resulting in 3 main categories and 7 sub-categories. While presented here sequentially, the coding process was circular, and emergent codes and sub-categories were compared in order to refine and re-code existing codes, in line with the principles of grounded theory analysis (Blaikie and Priest, 2019).

Two of the authors took the lead in the analysis (FK, ØJ), while the other author (TØ) acted as a co-analyzers of the coding and abstractions.

### 3.6 Strategies to achieve rigor in the study

We are aware of the potential researcher bias that could influence data collection and interpretation. We sought to reduce this bias by actively thinking reflexively throughout the research process and adopting different types of triangulation (method triangulation, investigator triangulation, and data source triangulation) (Denzin, 2017; Patton, 1999). To establish trustworthiness, we adopted the criteria created by Lincoln and Guba (1986). These criteria are credibility, transferability, dependability, and confirmability (Lincoln and Guba, 1986). Table 3 illustrates the strategies that were adopted in our study.

**Table 3:** Strategies adopted from Lincoln and Guba (1986).

Rigor criteria	Purpose	Original strategies	Strategies applied in our study
**Credibility**	To establish confidence that the results (from the perspective of the participants) are true, credible and believable.	Prolonged engagement; lengthy and intensive contact with the phenomena (respondents)	• Before the scheduled interview, we met or called all of our participants (except street-intercept) to introduce ourselves and engage participants in an informal conversation. • If we were unsure about something, we called our participants after the interview to get clarity on some thing they had mentioned in order to get the correct interpretation.
		Triangulation	• We applied two data collection approaches (dyadic and in-depth individual interview). • We used method triangulation, investigator triangulation, and data source triangulation.
		Peer debriefing	• We discussed the interview questions with nutritionists from three different regions of Norway. • Interviewing protocol reviews by academic and colleagues. • We had a regular project meeting with members of the project.
		Interviewing process and techniques	• Interviewing protocol tested at pilot interviews with older adults from a different region of Norway.
		Negative case analysis	• We include a wheelchair user and parent-child dyad.
**Dependability and confirmability**	To ensure the findings of this qualitative inquiry can be replicate if the inquiry occurred within the the same cohort of participants, coders and context. To increase the confidence that the results would be confirmed or corroborated by other researchers.	Rich description of the study methods	• A detailed description of the research procedures was provided, allowing others to conduct follow-up studies.
		Establishing an audit trail	• We have a detailed track record of the data collection process and all signed informed consent. • Audio files, transcripts, field notes writing, and reflection notes.
		Reflexivity	• Most of the time, two of the authors present during the interview process. • We keep reflection notes for each interview, allowing us to reflect not only on what happened at a particular interview but also on how we as researchers felt that day.
**Transferability**	The degree to which the results can be generalized or transferred to other contexts or settings.	Sampling	• We use maximum variation sampling in terms of participants' characteristics (see Tables 1& 2).
		Data saturation	• With dyadic interviews, we can learn about individual and collective perspectives, which adds dimension to the data collected. Furthermore, our sampling strategy allows us to identify more perceptions and variations. In doing so, the picture becomes broader and deeper to the point of saturation (Patton, 2002).

## 4 Results

**Fig. 1:**
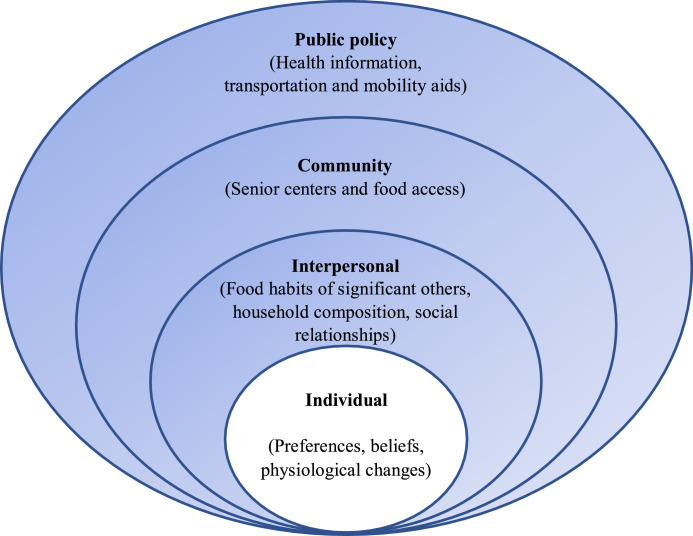
Environmental influences of eating behavior in home-living older adults. The ecological framework was adapted from McLeroy et al. (McLeroy et al., 1988). This study focuses on identifying specific environmental factors as depicted in the colored round figure.

The content analysis process described above resulted in three categories and seven sub-categories that play roles in older adult's eating behavior. Each of these categories and sub-categories is presented in more detail in the following section. An overview of the different categories and sub-categories is presented in Fig. 1, and examples of participants' quotes under each sub-category are given in Table 4.

**Table 4:** Example of supporting quotes.

Category	Sub-category	Examples of quotes
Interpersonal influences	Food habits of significant others	"It's just the two of us now, so we decide what we want to eat by looking in the fridge or freezer. My wife has control over that. First she asks me what I want to eat, and then she says she'd already decided, so I eat what she eats (laugh)." (P22)
		"I took great care of what my husband liked. I eat most things, but it often ended up with meat and potatoes. So I ate meat and potatoes too. Now that he is gone, I don't eat so much meat or potatoes. I eat more vegetables." (P3)
		"My ex-wife cooked, so I ate whatever she put on the table. Usually pasta or salad. Now I live alone most of the time, so I eat dinner at the cafeteria at work most of the time." (P6)
	Household composition	"I always make more, but then I freeze it. I have three in my family." (P14)
	Social relationship (friends and other relatives)	"I have almost no contact, but I have some girlfriends. We meet once a month at my place, and have dinner in an Indian restaurant, our neighbor. I don't go to the city center." (P8)
		" I have two daughters, one lives nearby, and one lives far away. She lives far away, but she comes and visits now and then and stays with me for one month. She helps me with the food shopping and takes care of everything else. At other times, some relatives nearby help when I need it." (P16)
Community influences	Senior Centers	"I've been here for two years. I am here because if I am not, I will be alone in my apartment looking at the wall. I need some social interaction with other people; I have no job, so I have no contact with other people. I have no siblings and very little contact with my family. The age difference is quite high here, but I don't mind because the people here are nice." (P8)
		"I'm here Monday and Tuesday, so the rest of the week, I make my food at home." (P13)
	Food access	" I decide what to eat by going to the shop because the shop is right under my place. I often go to the store, just buy different things I feel like eating, variations of what I feel like." (P6)
		"I have a cabin on an island, so I fish a lot. I prepare it as best I can." (P15)
		"Before they started building oil rigs in this fjord, the fjord was very clean. We eat a lot of fish; we eat whatever we catch." (P16).
Public policy	Health information	"I just heard about fat; they talked about it today, apparently now it is okay. It used to be a no-not, not okay, but now it is obviously okay. It's confusing." (P15)
		"I listen to the radio and talk to people, and everyone seems to be different, lots of information." (P13)
		"There is so much information about food and nutrition, but no one explains what information is correct." (P8)
	Transport and mobility aids	"I have rheumatism, so I can't walk so far, it's exhausting. I don't have a heavy-duty rollator, so I can't eat in that cafeteria" (P17)

**Category 1: Interpersonal influences (social environment)**

In terms of *interpersonal factors*, participants considered significant others' food habits and household size to be factors that affect the type and quantity of food they eat. Furthermore, support from relatives improves access to healthy food, and the interaction with their friends influences their eating habits when they are together.

### 4.1 Food habits of significant others

Most participants said they eat what they eat because of the people in their lives, and some discussed how they modified their diets in response to changes in the family.

"When the kids moved out, we never again ate tacos, pizza, tomato soup, fish sticks, or lasagna (laugh). We'd had enough of those dishes. When you have kids, you have little time available, so you tend to choose the safe choice so everyone can eat. But when they move out, we can choose freely. It becomes more varied and healthier (laugh)." (P1)

One participant mentioned that they converted to a vegan diet because of their daughter.

"What has affected our diet most in recent years is our daughter. Over the last few years, she has changed to a vegan diet, and that has affected me. Her partner is a vegan cook in a restaurant, so we learn a lot from them." (P5)

Others claimed that they have a vegetarian diet only when they are together with their partner.

"My girlfriend is vegetarian, but I don't eat vegetarian when she is not here. When she is here, we make dinner together." (P7)

### 4.2 Household composition

Participants talked about household composition as one factor that affects the portion size of food purchased and prepared at home. For participants living alone, they prefer to buy single-serving packages and smaller portions. In contrast, participants with two or more people in their households do not seem concerned with the portion size of food purchased or prepared at home.

"I chose the single-serving food because I am single. If I buy something with a bigger portion, I tend to eat more than I should." (P15)

"We bought so much food; our freezer is full. We haven't bought any food since Christmas." (P22)

Others living alone also mentioned preparing single-serving dinner because they don't like to eat the same leftovers for a few days.

"I only make my dinner for the day, one portion. I don't like to eat the same thing every day." (P12)

One other participant mentioned that he prefers to buy one-portion meals to avoid food waste.

"I used to make cucumber salad, but now if I make it, the rest goes in the garbage. I buy one portion if I want it instead." (P17)

### 4.3 Social relationship (friends and other relatives)

Social relationships are another essential factor that can influence the eating behavior of participants. Two social relationships that participants identified as conducive to a good diet were the relationships with friends and relatives. Participants reported that eating together with friends helps them establish connections and gives them a chance to learn more about food, and support from relatives ensures access to healthy food.

"Every week for almost 40 years, we have had a dinner club with the same people. It started before I had children, focus on good food. When the kid was small it was once a week. Lots of good food and exciting, we became very familiar with Italian cuisine." (P4)

Other participants mentioned how relatives help them with the grocery shopping make sure they have the food they need to stay well, and offer companionship regularly.

``Every Friday, me, my son, and his sons have coffee together, and then we shop for groceries. We buy enough food to set me up for one week, maybe even a little longer.'' (P10)

**Category 2: community influences (physical environment)**

The community where people live affects their access to healthy food and opportunities for engaging in healthy behavior (Brug et al., 2008). Participants described senior centers as a place that provides access to social interaction and nutritious food. Access to food is also considered to be an important contributor to maintaining a healthy diet.

### 4.4 Senior centers

The interviews revealed that older adults go to senior centers because they provide them with social interaction and food.

"Every Thursday, I go to the senior center. It's nice there, and there are so many people my age; we have been through the same things in life, so we talk and enjoy ourselves. They have concerts sometimes and there's lots to do when we are there. I look forward to it every week." (P3)

"The reason why I go to the senior center is because I get some food, food for three days. I deal with it the rest of the time on my own. It's not difficult. I buy ready-made food." (P16)

### 4.5 Food access

Participants reported that their food choice is often dependent on what is accessible. Some said they eat based on what is available in the season and the grocery store in their immediate neighborhood.

"What we eat is dependent a lot on the time of year, for example, barbecue food in the summer. We eat *Lutefisk* (a seafood dish) when it's that time of year. So, we eat according to the season." (P2)

"This time of year, my favorite is sheep with cabbage because lamb is in the shops at this time of year. Good lamb is available from the store nearby." (P10)

Others stated that they eat what they have grown in their garden due to convenient access and taste.

"We grow our vegetables in the garden, for example, Brussel sprouts. We can pick this up even in the winter when we want to cook dinner. Also, tomatoes, compared to the ones in the stores, they taste better." (P1)

Another participant mentioned that he has a farm, so he has access to meat.

"We have a farm, and we keep sheep, so we use lamb meat and don't have to buy it." (P14)

Furthermore, a few participants described how proximity to the sea allows them to enjoy more seafood.

``We have a cabin next to the sea, so we eat more fish and other seafood. We don't catch it ourselves; we buy it from the nearby fisherman.'' (P4)

**Category 3: Public policy (societal influences)**

When the participants were asked about government measures to support healthy eating among older people, most commented on health information while others discussed transport and mobility aids.

### 4.6 Health information

In all interviews, the participants were unanimous in the view that they were bombarded with health information. Participants reported confusion about what foods are healthy or what foods they should not eat, and anticipate that health advice will change.

"We read a lot. For example, we learn to cut out butter, then coffee is dangerous, and then coffee is no longer dangerous (laugh). We don't take it very seriously." (P2)

"So much information and alternative this and that, if you don't know what you need, how can you eat healthier, and why do you need to eat better." (P12)

### 4.7 Transport and mobility aids

One participant who has access to a facilitated transport card subsidized by the government raised an issue about the limited sum available on the card. The participant described difficulty getting to food shops and medical appointments due to transportation. Participants also expressed concern about mobility aids that help them move from place to place, such as from home to the grocery store or home to a cafeteria.

"We get very little on the transport card. You have to think about what you have to do all the time, like if you need to go to the shops to buy food, to the doctor, you have to use it. I have to go to the eye specialist sometimes, so I have to use that too. I have to calculate the little I have for transport, so I can't go out so much. It just doesn't help, you know." (P13)

"I try to pull myself together and walk down to the grocery store. Occasionally, the weather is awful, so you can't go. Going back home is even harder, up the hill. Soon I will have a heavy-duty rollator so that I can walk around. They have ordered it from the state welfare service, but I don't know when it will get here." (P3)

## 5 Discussion

The objective of this study was to explore environmental factors that influence home-living older adults' eating behavior. Discussions with participants led to the identification of environmental factors that facilitate and hinder healthy eating. Participants identified their significant others and the community where they live as the factors that have the greatest impact on their eating behavior and, to a lesser extent, their friends.

When asked about selecting what to eat, participants reported that their eating choices are often influenced by their significant others or people connected to them. In other words, who they dine with affects how they choose what to eat. This finding is supported by existing literature on the importance of social influences on eating behaviors (Higgs and Ruddock, 2020). Participants also reported help with food shopping from their relatives as an important aspect of healthy eating. As reported elsewhere, the household composition also affects older adults' food selection and purchasing decisions (Lesakova, 2016). For participants living alone, a smaller portion or single-serving packed food products is the preferred choice.

Outside of family, friends have some influence on the older adults' eating behavior. Although participants discussed their friends' role in impacting their eating behavior, it was not discussed as deeply as family influences. This finding likely reflects the study sample, which included more older adults living alone with a smaller social network than those living with a spouse, who have a more extensive social network. An older person's social network generally declines with age; this alteration could be because of the loss of a partner, retirement, failing health, cognitive impairment, or family members moving away or leaving home (McLaughlin et al., 2010). Having a good time and eating good food together was reported as the main reason for older adults having meals with friends.

Quite clearly, social aspects have a positive impact on eating behavior in older adults. Unfortunately, social contact and engagement often become less frequent with age (Stroebele-Benschop et al., 2016). Social facilitation of eating, such as having meals with others, may help older adults increase food intake and eat healthier.

In terms of the community where the participants live, senior centers emerged as an important physical environment that affects older adults' eating behavior. Participants described the senior center as a place where they can meet others to socialize, carry out some activities, and have a meal. As discussed above, older adults tend to have less social interaction than others. By offering opportunities for social interaction, senior centers can help reduce loneliness among older adults (Marquet et al., 2020). Participants also discussed how senior centers allow them to maintain their independence by providing food a few days a week. This finding would suggest that senior centers are one of the most important resources for the aging community. However, it is not necessarily the case that going to a senior center has positive benefits for all older adults. Senior centers serve diverse populations of older adults, which vary in age, health, and support needs. Also, the services offered at senior centers differ greatly, from social events to health services, so some programs are likely to only appeal to certain groups of the local older adults. One potential strategy to increase participation in senior centers is to offer a wide variety of leisure and social activities, as well as programs and services that promote healthy eating.

An additional factor influencing the food choice of older adults is access to food. For some participants, this includes convenient access to a grocery store, growing produce in the garden, farming, and living near the ocean. This finding suggests that food accessibility is a fundamental element of older adults' health (Kortright and Wakefield, 2011). Based on this, a community-driven program aimed at increasing access to affordable and nutritious food may help older adults improve their diet. Such programs can include special financing or tax incentives for grocery stores in rural areas, establishing farmers markets, food pantries, or meals on wheels.

What follows are the societal factors that influence the eating behavior of older adults. Societal influences seem to be more distal to older adults but can substantially affect them, their significant others, friends, and the community where they live. Factors within the societal level that can affect older adults' eating behavior are health information, transport support, and mobility aids.

Overall, our findings show that most older adults in this study reported confusion about health information and coming across conflicting information about proper diet and nutrition from different sources. This finding is consistent with the finding of Le et al. (2021), which showed that a significant proportion of the Norwegian population faces various challenges in dealing with health information. Furthermore, contradictory diet and nutrition information can undermine the success of the healthy eating promotion (Nagler, 2014). Thus, serious consideration should be given to reducing the ambiguity of diet and nutrition information. Ideally, scientists should create quality information, and the media then communicate it accurately to the public (Swire-Thompson and Lazer, 2020). Unfortunately, as well as providing opportunities for people to improve their eating behavior, the media also allows misinformation to flourish (Wang et al., 2019). Conflicting health information is a growing problem worldwide, and we are not well-positioned to help information seekers and healthcare professionals to manage this growing problem (Carpenter et al., 2016). Moving forward, we believe more research is needed to develop effective strategies to deal with conflicting health information.

As indicated previously, access to food is essential for older adults maintaining a healthy diet. Lack of access to transportation and mobility aids can in turn limit older adults' access to healthy food (Walker et al., 2010). Based on this finding, government support to increase subsidized transport and mobility aids can help improve older adults' dietary behavior. That said, this approach is likely to be more effective when integrated with other measures designed to promote healthy eating.

The environmental factors that affect the eating behavior of home-living older adults can be categorized into various levels of influence, as conceptualized by the ecological model: interpersonal (food habits of significant others, household composition, social relationships), community (senior centers and food access), and public policy (health information and transportation/mobility aids). The ecological model provides a useful framework for better understanding the multiple environmental factors that impact the eating behavior of home-living older adults. This model can therefore be used to guide research and can be applied in a study that focuses on modifying eating behavior. While the ecological model makes it possible to categorize the environmental factors that influence older adults' eating behavior, it lacks interaction within and between the different levels of influence (Sallis et al., 2015). Nevertheless, application of the ecological model looks promising for moving the field of health promotion closer to attaining the goal of improving the eating behavior of home-living older adults.

## 6 Limitations and future direction

Our study has successfully explored the influence of environmental factors on the eating behavior of home-living older adults. However, the limitations of the study, which included sample, findings, and scope, must be delineated.

The study involved only a small sample of Norwegian older adults. As such, the result of the study is limited to the selected participants and their eating behavior experiences. To reduce this challenge, we applied deliberate sampling for heterogeneity. Deliberate sampling for heterogeneity is recommended as the best alternative when random sampling cannot be used (Campbell, 1979; Shadish, 1993). This allowed us to look at sample members from all available angles, thereby achieving an in- depth understanding of the phenomenon. Additionally, the setting and cultural differences may also limit the transferability of the findings to a different context.

In terms of sample size, our intention was to have an adequate sample size. However, the adequate sample size needed for qualitative research findings to have some validity is difficult to estimate (Vasileiou et al., 2018). One way to improve the validity of our findings would have been to increase the number of participants. In the case of the dyadic interviews, this would have involved arranging more pairs to be interviewed. However, due to limited resources and time, we chose to sample heterogeneity instead of increasing the sample size. We postulated that such sampling would yield sufficient breadth and depth of the phenomenon being studied.

What follows is a description of the scope of this study. The study is limited in scope as the data were collected before the COVID-19 pandemic and does not therefore reflect the eating behavior of the participants during the pandemic. Prior to the pandemic, the vast majority of home-living older adults participated in social activities, such as attending senior centers and other community programs. However, all these services and programs were curtailed due to lockdown, quarantine, and social distancing measures. These public health measures related to COVID-19 are essential; however, these restrictions increase social isolation and the feeling of loneliness among older adults, which negatively impacts on the eating behavior of many older adults (Wu, 2020).

Despite these limitations, this study helps us understand the environmental factors that impact on home-living older adults' eating behavior and thus, contribute to the existing literature on eating behavior. This research has thrown up many questions awaiting further investigation. Further research should be undertaken to explore how COVID-19 has affected home-living older adults' eating behavior and the role of environmental factors in promoting or hindering healthy eating.

## 7 Conclusion

Improving dietary behavior and promoting healthier eating practices among home-living older adults will require a prolonged and more sustained effort that addresses not only individual influence but also environmental factors. The findings suggest that multiple social relationships influence older adults' eating behavior. Thus, the social environment of eating is likely an important factor in promoting healthy eating among home-living older adults. This study has also shown that several aspects of the local community need to be considered to improve the eating behavior of home-living older adults. Senior centers and accessibility to food are the main aspects of community influence that can affect home-living older adults' eating behavior. Continued efforts are needed to maintain well-being and promote healthy eating among the home-living older adults. At the societal level, strategies are required to reduce ambiguity in diet and nutrition information for older adults. There is also a need to ensure adequate mobility support for older adults living at home in order for them to maintain their independence and a healthy diet. Finally, adopting a single model as a basis for study and for developing strategies in promoting healthy eating is not considered optimal. Further research should incorporate ecological models with other modeling or theories.

Finally, as noted earlier, the data in this study were collected before the COVID-19 pandemic. This prevents us from drawing a conclusion about the role of environmental influences on eating behavior during the pandemic.

## Declaration of Competing Interest

The authors declare no potential conflicts of interest with respect to the research, the authorship, and/ or publication of this article.
